# Survival outcomes in patients with large (≥7cm) clear cell renal cell carcinomas treated with nephron-sparing surgery versus radical nephrectomy: Results of a multicenter cohort with long-term follow-up

**DOI:** 10.1371/journal.pone.0196427

**Published:** 2018-05-03

**Authors:** M. W. W. Janssen, J. Linxweiler, S. Terwey, S. Rugge, C.-H. Ohlmann, F. Becker, Ch. Thomas, A. Neisius, J. W. Thüroff, S. Siemer, M. Stöckle, F. C. Roos

**Affiliations:** 1 Department of Urology and Pediatric Urology, Saarland University Medical Center and Saarland University Faculty of Medicine, Homburg/S., Germany; 2 Boxberg Center for Urology, Neunkirchen, Germany; 3 Department of Urology and Pediatric Urology, University Medical Center of Johannes Gutenberg University, Mainz, Germany; Eberhard Karls University, GERMANY

## Abstract

**Background:**

Does the dogma of nephron sparing surgery (NSS) still stand for large renal masses? Available studies dealing with that issue are considerably biased often mixing imperative with elective indications for NSS and also including less malignant variants or even benign renal tumors. Here, we analyzed the oncological long-term outcomes of patients undergoing elective NSS or radical tumor nephrectomy (RN) for non-endophytic, large (≥7cm) clear cell renal carcinoma (ccRCC).

**Methods:**

Prospectively acquired, clinical databases from two academic high-volume centers were screened for patients from 1980 to 2010. The query was strictly limited to patients with elective indications. Surgical complications were retrospectively assessed and classified using the Clavien-Dindo-classification system (CDS). Overall survival (OS) and cancer specific survival (CSS) were analyzed using the Kaplan-Meier-method and the log-rank test.

**Results:**

Out of in total 8664 patients in the databases, 123 patients were identified (elective NSS (n = 18) or elective RN (n = 105)) for ≥7cm ccRCC. The median follow-up over all was 102 months (range 3–367 months). Compared to the RN group, the NSS group had a significantly longer median OS (p = 0.014) and median CSS (p = 0.04).

**Conclusions:**

In large renal masses, NSS can be performed safely with acceptable complication rates. In terms of long-term OS and CSS, NSS was at least not inferior to RN. Our findings suggest that NSS should also be performed in patients presenting with renal tumors ≥7cm whenever technically feasible. Limitations include its retrospective nature and the limited availability of data concerning long-term development of renal function in the two groups.

## Introduction

Beside the ongoing dispute about overtreatment for benign renal masses and strategies to avoid it [1–4], we ask what would happen if we would limit surgical intervention even in the large renal masses to just malignant cases. Would the dogma of nephron sparing surgery stand even for large malignant renal masses? Recently, Mir et al. warranted more studies to better define the role of Nephron-sparing surgery (NSS) in this challenging clinical scenario[5]. Nephron-sparing surgery (NSS) is accepted as gold standard treatment for cT1 renal masses [6–8]. A survival advantage for patients undergoing NSS compared to radical nephrectomy (RN) could be reported in several non-randomized retrospective studies [9–12]. In contrast, the only prospective randomized study including 250 patients for either surgical group with a median follow-up of nine years showed a survival advantage for the RN treated patients [13]. However, several multicenter studies have provided data showing the survival advantage of NSS over RN for patients presenting with pT1 RCC using propensity score matching in order to limit selection bias [8, 14–17]. Only very few studies have shown good oncological outcome with short follow-up conducted by a low comorbidity rate for elective NSS in selected patients presenting with renal tumors ≥7cm [12, 14]. According to these studies, very little is known about the long-term oncological outcome. The current available literature about NSS in large renal masses is often biased. Most studies investigating oncological outcome analyzed cohorts including different histological subtypes for RCC or even benign subtypes. Notably, no published study so far provides good comparability for elective NSS and RN according to incidentally diagnosed cT2 RCC. Our objectives were to compare the oncological outcome and the complication rate in a very well balanced cohort.

## Methods

Prospectively acquired databases (approved by the Local Ethics Committees, Aerztekammer des Saarlandes and Rheinhessen, Ref. number 188/05) of two academic urological referral centers (Saarland University Medical Center, Department of Urology, Homburg/Saar, Germany and Johannes Gutenberg University Medical Center, Department of Urology, Mainz, Germany), in total 8664 datasets, were searched for patients that underwent elective surgery (NSS or RN) for large (≥cT2) renal tumors. All datasets were fully anonymized before analysis.

The detailed criteria of the database query were defined as follows: renal masses of ≥7cm (≥cT2), time span from 1980 (start of nephron sparing surgery) to 2010 (start of robotic nephron sparing surgery, all laparoscopic/robotic cases were excluded to eliminate any learning curve effect), this first search revealed 1633 patients. This cohort was further limited by excluding patients with any benign tumor type in the final histo-pathological analysis, or imperative indication for NSS (as patients with previous renal surgery, known renal insufficiency, anatomic or functional solitary kidney). Using the radiological or surgical reports and reviewing the images wherever accessible, the tumor location was limited to exophytic growing tumors at the lower pole, the upper pole or the lateral margin of the kidney. Furthermore, any patient with centrally located masses, renal masses classified as “endophytic” or “multifocal”as reported in the final surgical documents and any T-stage >cT2 were excluded. Any patient with suspicious lymph nodes or metastatic disease at time of operation was excluded as well. To yield the maximum of oncological comparability between both groups any malignant mass other than clear cell renal carcinoma (ccRCC) was excluded. Three patients were excluded for insufficient data records. Finally, 123 patients were included. Patient´s results in terms of perioperative and oncological outcomes were first analyzed following the clinical stage obtained by imaging reports (radiographic findings in the preoperative CT scan) (n = 123). In the second analysis, the patient´s results were analyzed following the pathological stage and limited to tumors classified as pT2 in the final histopathological report (n = 83).

All patients included were staged preoperatively. Patients were selected for NSS according to tumor size and location. The decision to perform NSS was based on discussion and approval by interdisciplinary internal review boards (departments of urology and radiology at each center). Demographic and perioperative data, such as American Society of Anesthesiologists (ASA) score or the Clavien-Dindo-classification of surgical complications [18] were collected from patients´ medical records. Pathological tumor size was defined as the maximum diameter of the pathological specimen; tumor stage was revised according to the 2010 TNM classification system for all included cases. NSS and RN were performed using standard open surgical techniques as previously reported [19]^,^[6]. Perioperative data were obtained from the databases. Follow-up data, including cause of death and recurrence of malignancy for each patient, were actively obtained from patients´ general practitioners or the cancer-registries. Time to disease progression was defined as the interval between surgery and the appearance of new soft-tissue masses on radiologic imaging. The data are presented in median and range (for continuous variables) or as absolute and relative frequencies (for categorical variables). The clinic-pathological features were compared between the two groups using the Wilcoxon rank sum test, chi-square test and contingency test (Fisher´s exact test). To exclude an age difference between the groups, a matched-pair analysis allowing sampling with replacement was executed. Overall survival (OS) and cancer-specific survival (CSS) were estimated using the Kaplan-Meier-method; the log-rank (Mantel-Cox) test was used to compare survival among different groups. No propensity score matching or further univariate and multivariate Cox proportional hazard regression models could be applied due to the very small number of events in the study groups. Statistical analyses were performed using Statistical Package for GraphPad PRISM Version 7.01 (GraphPad Software, Inc. La Jolla, CA) and SPSS Version 17.0 (Chicago, IL). All tests were two-sided, *p*-values <0.05 were considered statistically significant. Because the present study was an exploratory study and no adjustment for multiple testing was done, the *p*-values are descriptive only.

To compare our results to the current literature a pubmed® search was performed. The first keywords used in the search were “partial versus radical nephrectomy OR nephron sparing” and revealed over 2000 hits, in a more detailed search the keywords were adjusted to “partial nephrectomy “OR” nephron sparing “AND” T2 “, and the search revealed 68 hits. Only relevant articles reporting functional and oncological outcomes were included.

## Results

Out of total 8664 patients in the two databases, the first query revealed 1633 patients with nephrectomy or NSS for large (≥cT2) renal masses, excluding all non-malignant cases and malignant cases other than ccRCC. Furthermore, all cases of cyto-reductive nephrectomy, revealing 681 patients and 43 patients treated by NSS for ccRCC were excluded. Excluding all cases with secondary tumors or recurrent tumors or imperative indications finally obtained 123 patients, who met the inclusion criteria outlined in the methods section in detail. Main patient characteristics, perioperative results as well as oncological follow-up data are listed in Table 1. A subgroup of 83 patients was classified as pT2 in the final histo-pathological report (NSS = 11 or RN = 72). Most demographic and clinical items did not differ significantly between the elective NSS and RN group. Only the significant complications graded as CDS ≥2 occurred more frequently in the NSS group (NSS vs. RN 44.4% vs. 31.5%; p = 0.039). Four Grade 3 CDS occurred overall. The majority of grade 2 complications represent blood transfusions.

**Table 1:** Patient demographics, and perioperative results stratified by surgery type for all diagnosed ccRCC.

	RN	NSS	*P* value
Patients	105	18	
Male sex (%)	67% (71/105)	50% (9/18)	n.s.
Age (y) (median, range)	62 (32/80)	57 (43/76)	n.s.
ASA overall			n.s.
ASA ≥3 (%)	23/105 (21.9%)	3/18 (16.6%)	n.s.
Tumour diameter (cm) (median and range)	8 (7.0/18.0)	8 (7.0/16)	n.s.
Pathological TNM result			
pT1b	5 (4.7%)	4 (22.2%)	
**pT2**	**72 (68.5%)**	**11 (61.1%)**	n.s.
**pT2a**	**50 (47.6%)**	**11**	0.032
**pT2b**	**22 (20.9%)**	**0**	
pT3	28 (26.6%)	3 (16.6%)	n.s.
Clavien Dindo score overall			n.s.
Clavien Dindo score ≥2	33/105 (31.5%)	8/18 (44.4%)	0.039
Clavien Dindo score ≥3	1/105 (1%) Severe pulmonary embolism	3/18 (16.7%) 2x acute bleeding, 1x urinoma,	0.007
Timespan OS (months) (median,/range)	93 (3/367)	163 (3/296)	n.s.
Timespan CSS (months) (median/range)	87 (3/367)	163 (11/296)	n.s.

Note: ASA: American Society of Anesthesiologists score (ASA)[36] NSS: nephron spearing surgery RN: radical nephrectomy

According to the current TNM classification, the pathological workup resulted in an up-staging from cT2 to pT3a in 16.3% and 26.6%, and in a down-staging from cT2 to pT1b in 22.2% and 4.7% of patients in the NSS and RN group, respectively.

The pT2 tumors were further subdivided into pT2a (n = 50, 47.6%) and pT2b (n = 22, 21.0%) for the RN group. In the NSS group, all patients were pT2a. The median follow-up was 102 months (range 3–367 months) for all patients, 163 months (range 3–296) in the NSS group, and 93 months (range 3–367) in the RN.

During follow-up, five (27.7%) and 61 (58.1%) patients died after NSS and RN, respectively as listed in Table 2. Among the five patients in the NSS group two of them had a pT3a ccRCC. One patient died from metastatic ccRCC, of the remaining four, two died from different cancers (leukemia and lung cancer) and two died from cardiovascular disease. Among the 61 patients in the RN group, 38 (36.2%) patients died due to ccRCC. Of these, 28 had been diagnosed with a pT2 and nine with a pT3a ccRCC. 23 patients (21.9%) died not from renal cancer-related causes, namely from cardiovascular disease in 7 cases, from other or unknown reasons in 14 cases and from other cancers in two cases (lung and ovarian cancer).

**Table 2:** Patients who died or suffer from recurrence of disease during follow-up stratified by surgery type for all diagnosed ccRCC.

	RN	NSS	*P* value
**Death during follow-up**	**61/105 (58.1%)**	**5/18 (27.7%)**	**n.s.**
Cause of death “RCC”	38/105 (36.2%)	1/18 (6%)	n.s.
Other cause of death “other cancer”	2/105 (1.9%)	2/18 (11.1%)	n.s.
Other cause of death “cardio/vascular”	7/105 (6.7%)	2/18 (11.1%)	n.s.
Other cause of death “unknown” or other	14/105 (13.3%)	0	
Cause of death “non malignant”	23/105 (21.9%)	2/18 (11.1%)	n.s.
Patients with recurrence of disease (all)	42/105 (40%)	2/18 (11.1%)	0.01
…only pT1b and pT2	30/77 (38.9%)	1/15 (6%)	0.016
…only pT2	30/72 (41.6%)	1/ 11 (9%)	0.046
Patient with local recurrence/metastases under therapy	4 (3.8%)	1 (5.5%)	n.s.

**Fig 1:**
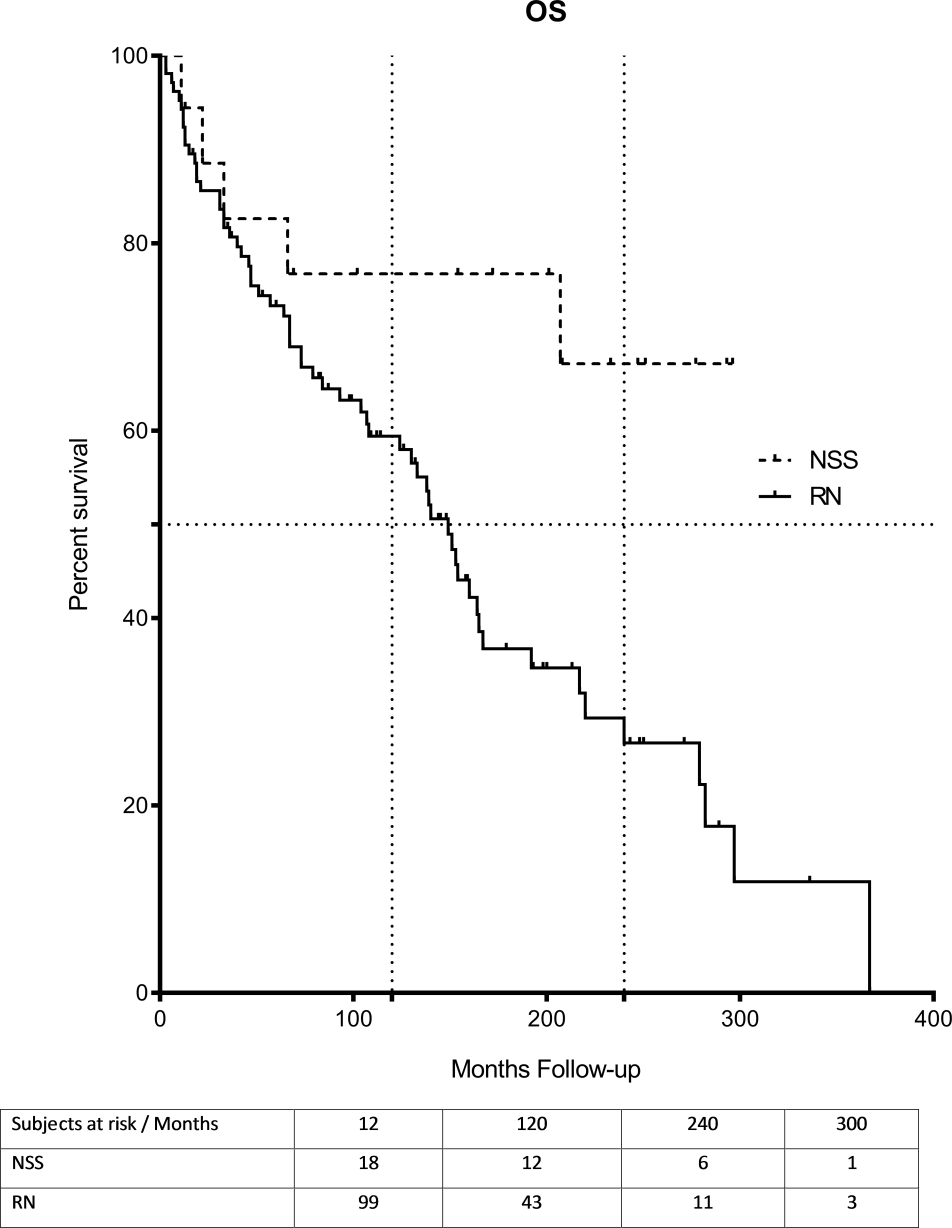
Overall survival (OS). OS for 123 patients cT2 after radical nephrectomy (RN, n = 105) or elective NSS (n = 18) for ccRCC ≥7cm. Comparison of survival analysis performed using log-rank (Mantel-Cox) test; Chi square 5.94, *P =* 0.014. Median OS was 149 months (range 3–367), for NSS and was not reached for NSS.

**Fig 2:**
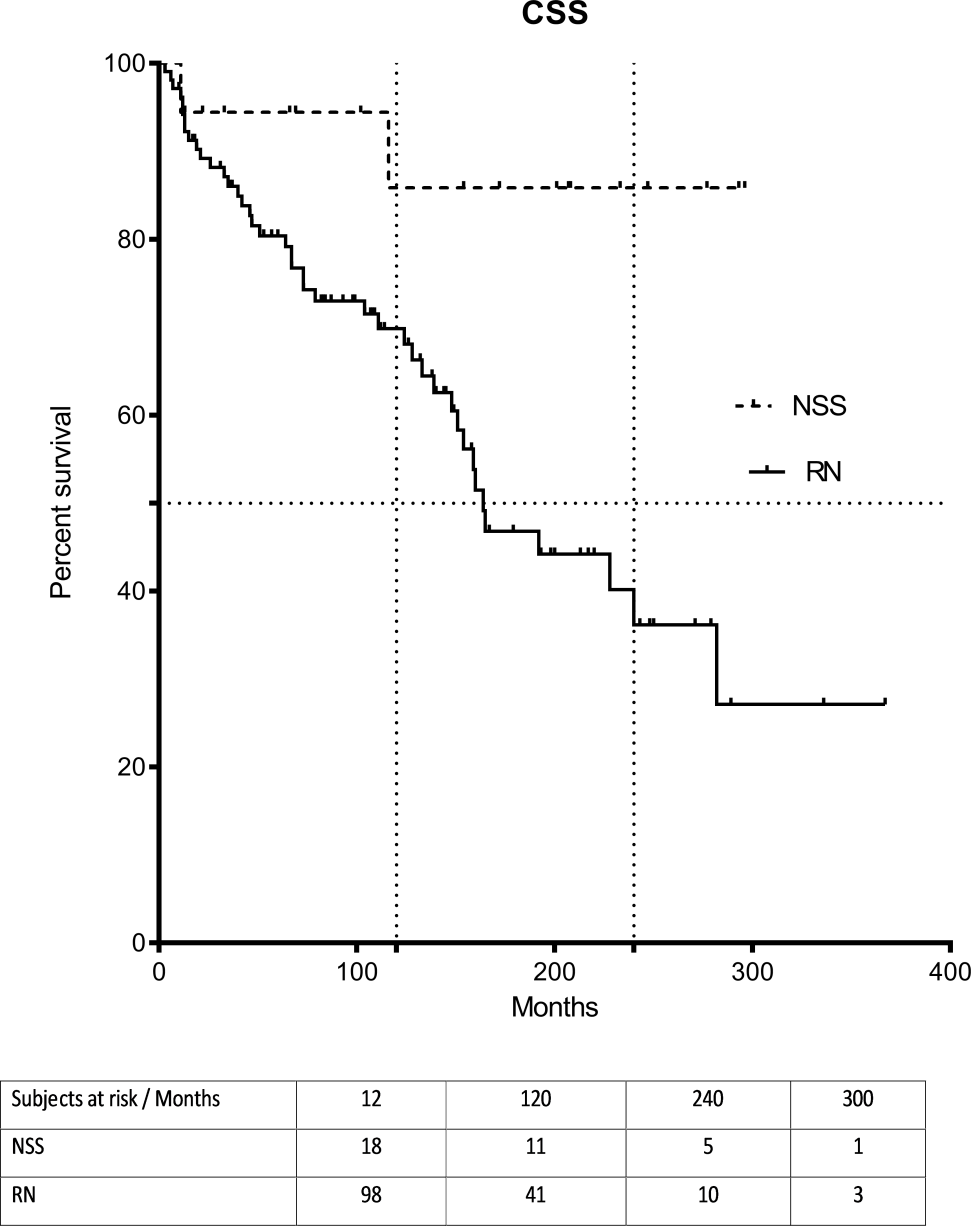
Cancer-specific survival (CCS). CSS for 123 patients cT2 after radical nephrectomy or elective NSS for ccRCC ?7cm. Comparison of survival analysis performed using log-rank (Mantel-Cox) test; Chi square: 3.35, P = 0.04. Median survival for RN was 164 months (range 3–367 months), for NSS not reached.

Local recurrence or distant metastases occurred in 5.5% (1/18) of patients in the NSS group and in 3.8% (4/105) of patients in the RN group. Of these five patients, four were treated surgically for local or distant recurrence and one patient started systemic therapy with a tyrosine kinase inhibitor. A total of 57 patients of both groups are still alive including all five patients that suffered from local recurrence or metachronous distant metastases. Kaplan-Meier analysis showed significant differences in OS (p = 0.014) and CCS (p = 0.036) between the NSS and RN groups in favor of the former (Figs 1 and 2). Median OS and CSS were not reached in NSS group and were 164 and 149 months in the RN group. There was a significant difference in OS (p = 0.004) and CSS (p = 0.015) for the subgroup pT2 patients as well (S1 and S2 Figs). Here the 10- and 20-year CSS rates were 74.6% and 38.4% for RN and the 10-years CSS rate for the NSS treated patients was 88.9% estimated (S1 Fig and S2 Fig).

The comparison of OS and CSS for pT2a vs. pT2b patients in the RN group showed no significant differences (S3 and S4 Figs). After matching the study groups for age at operation in a 2:1 ratio for RN versus NSS, the Kaplan-Meier analysis showed no significant difference in OS and CSS (S5 Fig and S6 Fig). The current literature about NSS in large renal masses was reviewed and is compared in relevant topics in Tables 3 and 4.

**Table 3:** Overview of the current literature, comparing studies with patients presenting pT2 and cT2 RCC and treated by nephron sparing surgery (NSS) or radical nephrectomy (RN). Synopsis current literature.

Study	Study period	Number of centers	NSS	RN	Histology	Follow-up (months)
**Margulis et al. #(22)**	1990–2006	1	34 (73% imperative NSS)	567	pT2-T3b all different subtypes of RCC	62.1 (mean)
**Jeldres et al. #(27)**	1984–2001	13	29 (no statement about imperative indication)	896	pT1-T3 (T3 = 52.7%) different subtypes of RCC	40.8 (median)
**Breau et al.** **#(26)**	1970–2008	1	69 (13% imperative)	207	pT2-pT3b (13% pT3b) different subtypes of RCC,	38.4 (median)
**Long et al. #(25)**	n.a.	1	46 (45.9% with absolute or imperative indication for NSS)	-	pT2 and T3a (6.2% benign renal masses)	13.1 (median)
**Koop et al. #(30)** **(Clinical stage T2)**	2002–2012	2	80 25% imperative indication	122	pT1- pT4 (3.3% benign renal masses)	41.5 (median)
present study*	1980–2010	2	18	123	pT1b-pT3a only ccRCC only elective	102 (median)

**Table 4:** Overview of the recurrence and survival of the current literature, comparing studies with patients presenting pT2 and cT2 RCC and treated by nephron sparing surgery (NSS) or radical nephrectomy (RN).

Study	Recurrence NSS vs. RN		Survival			
			CSS		OS	
			NSS	RN	NSS	RN
**Margulis et al. #(22) **	4/34 (11.7%)	164/567 (28.9%)	5-year CSS (p = 0.1)		n.a.(not applicable)	
			78 ±10%	74±3%		
**Jeldres et al. #(27)**	n.a.	n.a.	5-year CSS		Only cause specific survival NSS associated with 5.3-fold higher rate of cancer–specific mortality	
			67.0%	87.2%		
**Breau et al.** **#(26)**	19/69 (27.5%)	76/207 (36.7%)	Event at median follow-up (3.2 years)		Death at median follow-up (3.2 years)	
			12/69 (17%)	53/207 (25%)	27/69 (39%)	88/207 (43%)
**Long et al. #(25)**	5 (10.9%)	n.a.	94% Overall and CCS at 5 years			
			70.9% Overall and CSS at 10 years			
**Koop et al. #(30)** **(Clinical stage T2)**	Only 5-year PFS (p = n.s.) 79.9% vs. 69.8%		5-year CSS only (p = n.s.)		5-year OS only (p = n.s.)	
			86.7%	82.5%	83%	80%
present study*	2/18 (11.1%)	42/105 (40%)	5-year CSS		5-year OS	
			94.4 ± 5.4%	80.4 ±4.1%	82.6% ±9.1%	73.3± 4.4%
			10-year CSS		10-year OS	
			85.9 ± 9.5%	69.8 ±5.0%	76.7%±10.2%	59.4 ±5.1%

## Discussion

To our best knowledge, the present study reports the longest documented oncological follow-up, comparing elective NSS to RN in patients presenting with pT2 ccRCC.

Several risk factors for worse pathological outcome in these tumors, e.g. higher tumor grade, more tumor necrosis, and higher T stages, were reported previously [20]. Therefore, the expansion for elective NSS in larger RCC (>7cm) might be both, surgical and pathological challenging. In order to minimize any bias, negative prognosticators were excluded from our analysis. Furthermore, the access to NSS was limited to tumors growing exophytically from the upper / lower pole or lateral margin of the kidney. The recurrence of disease rate in the NSS group of our cohort is in line with the findings reported by Margulis et al.[21] and others [22–24]; the higher recurrence rates observed in the RN group may due to the higher selection criteria in our cohort. Recently, Mir et al. conducted a meta-analysis to compare NSS and RN and could only find four published series for pT2 and cT2 disease [5] (detailed comparison in Table 3 and Table 4 at the end of this manuscript). Interestingly, they found a lower recurrence rate and cancer-specific mortality in the NSS group, but no significant difference in all-cause mortality. They concluded that NSS offers the same cancer control and potentially better long-term survival due to the preservation of renal function. After adjustment for age, the results of the present study (S5 Fig and S6 Fig) are in line with the above mentioned analysis. But overall, we demonstrated a significant OS benefit after NSS (Fig 1). Very few studies addressed elective NSS for pT2 or higher rated renal tumors [12, 14, 24–28]. Jelders et al. included only 29 NSS patients, as Breau et al. included only very few patients undergoing elective NSS in their cohorts, both groups did not provide any information about the imperative indication of NSS [25, 26].

In the study by Margulis et al., only 27% of the NSS patients had elective indication for NSS[21]. These findings underline the unique character of the here presented data. Besides that, another point to consider is that all above mentioned studies included different RCC subtypes, up to 26% papillary (Breau et al.) and 15% (Margulis et al.) or 13,8% (Jeldres et al.) chromophobe RCC. More recently, Kopp´s series of 202 patients with clinical stage 2 (≥7cm) renal masses consisted of 12,7% benign cases in the NSS-group. This may bias their conclusion, that there would be a way to identify patients with T2 renal masses who could benefit most from NSS, and may identify a group of patients in elective circumstances, where NSS would not yield significant benefit but exposes the patient to increased risks [29]. Interestingly, in the histopathological results they found—in concordance to our results—an upstaging in 11–15% to pT3 or even pT4 tumors and a down-staging up to 4,3% to pT1 tumors in the NSS group.

For analyzing oncological outcome for patients with pT2 RCC, we excluded patients with pT3 RCC. The analysis of OS revealed a 50% survival advantage after 20 years for patients treated by NSS (S1 Fig).

Our study was neither biased by less malignant or even benign renal lesions nor by negative prognosticators [30]. Our selection criteria elucidate the natural history of RN or NSS for large ccRCC. We excluded minimal-invasive cases from our series in order not to bias our study with learning-curve effects from the upcoming minimal-invasive or robotic approaches. Nevertheless the later mentioned approaches have already lead to safe and equivalent oncological results in even more complex renal masses [31].

Interestingly, the sub-classification of our RN group into pT2a and pT2b showed no significant difference for OS (*p* = 0.23) or CSS (*p* = 0.57) (S3 Fig and S4 Fig). This finding is in line with other publications and questions the necessity of the pT2 sub-classification into pT2a and pT2b.

We hypothesize, that good selection parameters, as exophytic masses limited to the upper or lower pole, should allow the surgeon to offer patients equivalent oncological control with improved OS.

To offer the same surgical results it is important to mention that NSS in larger tumors are correlated to increased perioperative morbidity. Nevertheless Stephenson et al. could not show significant differences in morbidity in between radical and nephron sparing surgery groups On contrary in our study more CDS grade III complications were found in the NSS group, leading to a significantly higher re-intervention rate of 2.5% for NSS as compared to 0.6% for RN (p = 0.002)[32]. Patard et al. reported complication rates for NSS in pT2 or higher tumours: mainly urinoma (17.9%) in 40 patients with imperative indications. CDS grade 3 and 4 complications occurred in 20% [33]. In the meta-analysis by Mir et al., the risk ratio clearly favors RN, but they conclude that, NSS is a feasible approach despite the better perioperative morbidity in RN [5]. The nephrometric scoring systems like R.E.N.A.L or PADUA do help to provide more comparability about the complexity of renal masses in between study groups [34, 35]. A limitation of our study is that we were not able to provide such data retrospectively for our dataset due to only limited access to the involved images of older cases. One might discuss that only less complex tumors were chosen for NSS, to limit this drawback we allowed only exophytic tumors in both groups according to the final surgical reports.

The low rate of complications in the present study can be explained by the highly selective patient cohort, and again underlines the impact of careful patient selection and surgical experience. The presented cohort is well balanced; no significant differences in age, gender distribution or ASA scoring were found, as presented in Table 1. Though the overall complication rate did not differ significantly between the NSS and the RN group, severe complications (graded CDS III) occurred more frequently in the NSS group Table 1. Therefore, even in experienced centers the risk of complications performing NSS is not to be neglected. The percentage of cardiovascular related deaths did not differ significantly between the two groups as outlined in Table 2. This result might be biased by the higher rate of cancer-specific deaths in the RN group, but due to the small sample size it is not appropriate to perform further statistical tests in the presented cohort. A second limitation is the missing detailed information about the used clamping technique and follow-up about the renal function throughout the whole follow-up period. Since different clamping techniques were used und different protocols about renal preconditioning for renal ischemia were used, we cannot provide this data. Nevertheless, only nine out of 61 patients who died during follow-up died due to cardiovascular reasons that may be related to deteriorated renal function.

## Conclusion

Due to the small number of patients and events any conclusion must be made with caution, nevertheless due to the highly selected and well balanced cohort we conclude that elective NSS for ccRCC ≥7cm seems to be technically feasible with acceptable complication rates. Important to mention are the highly trained surgeons, technical attention, and very careful patient selection. The upcoming era of robotic assisted renal surgery may influence operative results significantly. The median follow-up period was rather long but included only a limited number of events which may obscure the good oncologic outcomes.

## Supporting information

S1 Fig Overall survival (OS) pT2 patients only. Overall survival (OS) for 83 patients after radical nephrectomy (RN) or elective Nephron-Sparing Surgery (NSS) sub-classified retrospectively according to the 2010 TNM classification into pT2. Comparison of survival analysis performed using log-rank (Mantel-Cox) test; Chi square: 8,30, *P =* 0.004. Median survival for pT2 in RN group was 149 months (range 3–367 months), not reached for NSS group.
(TIF)

S2 Fig Cancer-specific survival (CSS) for pT2 patients only. CSS for 83 patients after radical nephrectomy (RN) or NSS sub-classified retrospectively according to the 2010 TNM classification into pT2. Comparison of survival analysis performed using log-rank (Mantel-Cox) test; Chi square: 5,866, *p =* 0,015. Median CSS for pT2 was 164 months (range 3–367 months) for RN, not reached for NSS.
(TIF)

S3 Fig Overall survival for subclassified pT2a vs. pT2b. OS for 72 patients after radical nephrectomy (RN) sub-classified into pT2a (n = 50) vs. pT2b (n = 22) retrospectively according to the 2010 TNM classification. Comparison of survival analysis performed using log-rank. Chi square 1.4, *P =* 0.2. Median OS was 149 months (range 6–289) for pT2a, and 164 months (range 7–336) for pT2b. Hazard ratio (log-rank) 1.46 (95% CI 0.79 to 2.68) revealed no significant difference.
(TIF)

S4 Fig Cancer specific survival for subclassified pT2a vs. pT2b. CSS for 72 patients after radical nephrectomy (RN) sub-classified into pT2a vs. pT2b retrospectively according to the 2010 TNM classification. Comparison of survival analysis performed using log-rank (Mantel-Cox) test; Chi square 0.80, *P* = 0.3. In Median CSS was 160 months (range 6–289) for pT2a, and 165 months (range 7–336) for pT2b. Hazard ratio (logrank) 1.41 (95% CI 0.67 to 2.95) no significant difference was demonstrated.
(TIF)

S5 Fig Overall survival (OS) for age mateched patients. OS for 54 age-machted patients, for matching sampling with replacement was allowed, patients machted 2:1 for radical nephrectomy (RN) and nephron sparing surgery (NSS), respectively. Comparison of survival analysis performed using log-rank (Mantel-Cox) test; Chi square 2.3, *P* = 0.12. Median OS was 217 months (range 18–367) for RN and not reached for NSS. Hazard ratio (logrank) 2.05 (95% CI 0.89 to 4,69) no significant difference.
(TIF)

S6 Fig Cancer specific survival for aged mateched patients. CCS for 54 age-machted patients, for matching sampling with replacement was allowed, patients were machted 2:1 for radical nephrectomy (RN) and nephron sparing surgery (NSS), respectively. Comparison of survival analysis performed using log-rank (Mantel-Cox) test; Chi square 3.16, *P* = 0,07. Median CSS was 240 months (range 19 to 336 months) for RN and not reached for NSS. Hazard ratio (logrank) 3.49 (95% CI 1.21 to 10.1) no significant difference.
(TIF)

## Abbreviations

NSS: nephron sparing surgery
RN: radical nephrectomy
ccRCC: clear cell renal cell carcinoma

## References

[1] Kutikov A, Uzzo RG, Smaldone MC, Haifler M, Bratslavsky G, Leibovich BC. Reply to Patrick O. Richard, Micheal A.S. Jewett and Antonio Finelli's Letter to the Editor re: Alexander Kutikov, Marc C. Smaldone, Robert G. Uzzo, Miki Haifler, Gennady Bratslavsky, Bradley C. Leibovich. Renal Mass Biopsy: Always, Sometimes, or Never? Eur Urol 2016;70:403–6. Eur Urol. 2017;71(2):e47–e8. doi: 10.1016/j.eururo.2016.07.011 . 27436159 10.1016/j.eururo.2016.07.011

[2] Kutikov A, Smaldone MC, Uzzo RG, Haifler M, Bratslavsky G, Leibovich BC. Renal Mass Biopsy: Always, Sometimes, or Never? Eur Urol. 2016;70(3):403–6. doi: 10.1016/j.eururo.2016.04.001 . 27085625 10.1016/j.eururo.2016.04.001

[3] Jewett MA, Mattar K, Basiuk J, Morash CG, Pautler SE, Siemens DR, et al Active surveillance of small renal masses: progression patterns of early stage kidney cancer. Eur Urol. 2011;60(1):39–44. doi: 10.1016/j.eururo.2011.03.030 . 21477920 10.1016/j.eururo.2011.03.030

[4] Haifler M, Kutikov A. Current Role of Renal Biopsy in Urologic Practice. Urol Clin North Am. 2017;44(2):203–11. doi: 10.1016/j.ucl.2016.12.006 . 28411912 10.1016/j.ucl.2016.12.006

[5] Mir MC, Derweesh I, Porpiglia F, Zargar H, Mottrie A, Autorino R. Partial Nephrectomy Versus Radical Nephrectomy for Clinical T1b and T2 Renal Tumors: A Systematic Review and Meta-analysis of Comparative Studies. Eur Urol. 2017;71(4):606–17. doi: 10.1016/j.eururo.2016.08.060 . 27614693 10.1016/j.eururo.2016.08.060

[6] Novick AC, Streem S, Montie JE, Pontes JE, Siegel S, Montague DK, et al Conservative surgery for renal cell carcinoma: a single-center experience with 100 patients. J Urol. 1989;141(4):835–9. . 2926874 10.1016/s0022-5347(17)41026-3

[7] Van Poppel H, Becker F, Cadeddu JA, Gill IS, Janetschek G, Jewett MA, et al Treatment of localised renal cell carcinoma. Eur Urol. 2011;60(4):662–72. doi: 10.1016/j.eururo.2011.06.040 . 21726933 10.1016/j.eururo.2011.06.040

[8] Ljungberg B, Bensalah K, Canfield S, Dabestani S, Hofmann F, Hora M, et al EAU guidelines on renal cell carcinoma: 2014 update. Eur Urol. 2015;67(5):913–24. doi: 10.1016/j.eururo.2015.01.005 . 25616710 10.1016/j.eururo.2015.01.005

[9] Van Poppel H. Efficacy and safety of nephron-sparing surgery. Int J Urol. 2010;17(4):314–26. doi: 10.1111/j.1442-2042.2010.02482.x . 20409229 10.1111/j.1442-2042.2010.02482.x

[10] Thompson RH, Siddiqui S, Lohse CM, Leibovich BC, Russo P, Blute ML. Partial versus radical nephrectomy for 4 to 7 cm renal cortical tumors. J Urol. 2009;182(6):2601–6. doi: 10.1016/j.juro.2009.08.087 ; PubMed Central PMCID: PMCPMC4171846. 19836797 10.1016/j.juro.2009.08.087 PMC4171846

[11] Weight CJ, Larson BT, Fergany AF, Gao T, Lane BR, Campbell SC, et al Nephrectomy induced chronic renal insufficiency is associated with increased risk of cardiovascular death and death from any cause in patients with localized cT1b renal masses. J Urol. 2010;183(4):1317–23. doi: 10.1016/j.juro.2009.12.030 . 20171688 10.1016/j.juro.2009.12.030

[12] Roos FC, Brenner W, Muller M, Schubert C, Jager WJ, Thuroff JW, et al Oncologic long-term outcome of elective nephron-sparing surgery versus radical nephrectomy in patients with renal cell carcinoma stage pT1b or greater in a matched-pair cohort. Urology. 2011;77(4):803–8. doi: 10.1016/j.urology.2010.09.020 . 21145093 10.1016/j.urology.2010.09.020

[13] Van Poppel H, Da Pozzo L, Albrecht W, Matveev V, Bono A, Borkowski A, et al A prospective, randomised EORTC intergroup phase 3 study comparing the oncologic outcome of elective nephron-sparing surgery and radical nephrectomy for low-stage renal cell carcinoma. Eur Urol. 2011;59(4):543–52. doi: 10.1016/j.eururo.2010.12.013 . 21186077 10.1016/j.eururo.2010.12.013

[14] Becker F, Roos FC, Janssen M, Brenner W, Hampel C, Siemer S, et al Short-term functional and oncologic outcomes of nephron-sparing surgery for renal tumours >/ = 7 cm. Eur Urol. 2011;59(6):931–7. doi: 10.1016/j.eururo.2011.02.017 . 21371812 10.1016/j.eururo.2011.02.017

[15] Simone G, Tuderti G, Anceschi U, Papalia R, Ferriero M, Misuraca L, et al Oncological outcomes of minimally invasive partial versus minimally invasive radical nephrectomy for cT1-2/N0/M0 clear cell renal cell carcinoma: a propensity score-matched analysis. World J Urol. 2016 doi: 10.1007/s00345-016-1923-2 . 27578234 10.1007/s00345-016-1923-2

[16] Takagi T, Kondo T, Iizuka J, Omae K, Kobayashi H, Yoshida K, et al Comparison of survival rates in stage 1 renal cell carcinoma between partial nephrectomy and radical nephrectomy patients according to age distribution: a propensity score matching study. BJU Int. 2016;117(6B):E52–9. doi: 10.1111/bju.13200 . 26073989 10.1111/bju.13200

[17] Chung JS, Son NH, Lee SE, Hong SK, Lee SC, Kwak C, et al Overall survival and renal function after partial and radical nephrectomy among older patients with localised renal cell carcinoma: a propensity-matched multicentre study. Eur J Cancer. 2015;51(4):489–97. doi: 10.1016/j.ejca.2014.12.012 . 25576517 10.1016/j.ejca.2014.12.012

[18] Dindo D, Demartines N, Clavien PA. Classification of surgical complications: a new proposal with evaluation in a cohort of 6336 patients and results of a survey. Ann Surg. 2004;240(2):205–13. doi: 10.1097/01.sla.0000133083.54934.ae ; PubMed Central PMCID: PMCPMC1360123. 15273542 10.1097/01.sla.0000133083.54934.ae PMC1360123

[19] Robson CJ, Churchill BM, Anderson W. The results of radical nephrectomy for renal cell carcinoma. J Urol. 1969;101(3):297–301. . 5765875 10.1016/s0022-5347(17)62331-0

[20] Ficarra V, Secco S, Fracalanza S, Novara G, Gidaro S, Cindolo L, et al Expanding indication for elective nephron-sparing surgery in renal cell carcinoma. Arch Ital Urol Androl. 2009;81(2):86–90. . 19760862

[21] Margulis V, Tamboli P, Jacobsohn KM, Swanson DA, Wood CG. Oncological efficacy and safety of nephron-sparing surgery for selected patients with locally advanced renal cell carcinoma. BJU Int. 2007;100(6):1235–9. doi: 10.1111/j.1464-410X.2007.07225.x . 17979923 10.1111/j.1464-410X.2007.07225.x

[22] Iizuka J, Kondo T, Hashimoto Y, Kobayashi H, Ikezawa E, Takagi T, et al Similar functional outcomes after partial nephrectomy for clinical T1b and T1a renal cell carcinoma. Int J Urol. 2012;19(11):980–6. doi: 10.1111/j.1442-2042.2012.03085.x . 22735049 10.1111/j.1442-2042.2012.03085.x

[23] Jang HA, Kim JW, Byun SS, Hong SH, Kim YJ, Park YH, et al Oncologic and Functional Outcomes after Partial Nephrectomy Versus Radical Nephrectomy in T1b Renal Cell Carcinoma: A Multicenter, Matched Case-Control Study in Korean Patients. Cancer Res Treat. 2016;48(2):612–20. doi: 10.4143/crt.2014.122 ; PubMed Central PMCID: PMCPMC4843725. 26044158 10.4143/crt.2014.122 PMC4843725

[24] Long CJ, Canter DJ, Kutikov A, Li T, Simhan J, Smaldone M, et al Partial nephrectomy for renal masses >/ = 7 cm: technical, oncological and functional outcomes. BJU Int. 2012;109(10):1450–6. doi: 10.1111/j.1464-410X.2011.10608.x . 22221502 10.1111/j.1464-410X.2011.10608.x

[25] Breau RH, Crispen PL, Jimenez RE, Lohse CM, Blute ML, Leibovich BC. Outcome of stage T2 or greater renal cell cancer treated with partial nephrectomy. J Urol. 2010;183(3):903–8. doi: 10.1016/j.juro.2009.11.037 . 20083271 10.1016/j.juro.2009.11.037

[26] Jeldres C, Patard JJ, Capitanio U, Perrotte P, Suardi N, Crepel M, et al Partial versus radical nephrectomy in patients with adverse clinical or pathologic characteristics. Urology. 2009;73(6):1300–5. doi: 10.1016/j.urology.2008.08.492 . 19376568 10.1016/j.urology.2008.08.492

[27] Kim SH, Joung JY, Seo HK, Lee KH, Chung J. Baseline Chronic Kidney Disease and Ischemic Method of Partial Nephrectomy Are Important Factors for the Short- and Long-Term Deterioration in Renal Function for Renal Cell Carcinoma Staged T1-T2: A Retrospective Single Center Study. Biomed Res Int. 2016;2016:5398381 doi: 10.1155/2016/5398381 ; PubMed Central PMCID: PMCPMC5198085. 28074187 10.1155/2016/5398381 PMC5198085

[28] Alanee S, Herberts M, Holland B, Dynda D. Contemporary Experience with Partial Nephrectomy for Stage T2 or Greater Renal Tumors. Curr Urol Rep. 2016;17(1):5 doi: 10.1007/s11934-015-0558-y . 26715221 10.1007/s11934-015-0558-y

[29] Kopp RP, Mehrazin R, Palazzi KL, Liss MA, Jabaji R, Mirheydar HS, et al Survival outcomes after radical and partial nephrectomy for clinical T2 renal tumours categorised by R.E.N.A.L. nephrometry score. BJU Int. 2014;114(5):708–18. doi: 10.1111/bju.12580 . 24274650 10.1111/bju.12580

[30] Shah PH, Moreira DM, Okhunov Z, Patel VR, Chopra S, Razmaria AA, et al Positive Surgical Margins Increase Risk of Recurrence after Partial Nephrectomy for High Risk Renal Tumors. J Urol. 2016;196(2):327–34. doi: 10.1016/j.juro.2016.02.075 . 26907508 10.1016/j.juro.2016.02.075 PMC9235535

[31] Abdel Raheem A, Alatawi A, Kim DK, Sheikh A, Alabdulaali I, Han WK, et al Outcomes of high-complexity renal tumours with a Preoperative Aspects and Dimensions Used for an Anatomical (PADUA) score of >/ = 10 after robot-assisted partial nephrectomy with a median 46.5-month follow-up: a tertiary centre experience. BJU Int. 2016;118(5):770–8. doi: 10.1111/bju.13501 . 27102977 10.1111/bju.13501

[32] Stephenson AJ, Hakimi AA, Snyder ME, Russo P. Complications of radical and partial nephrectomy in a large contemporary cohort. J Urol. 2004;171(1):130–4. doi: 10.1097/01.ju.0000101281.04634.13 . 14665860 10.1097/01.ju.0000101281.04634.13

[33] Patard JJ, Shvarts O, Lam JS, Pantuck AJ, Kim HL, Ficarra V, et al Safety and efficacy of partial nephrectomy for all T1 tumors based on an international multicenter experience. J Urol. 2004;171(6 Pt 1):2181–5, quiz 435. . 15126781 10.1097/01.ju.0000124846.37299.5e

[34] Kutikov A, Uzzo RG. The R.E.N.A.L. nephrometry score: a comprehensive standardized system for quantitating renal tumor size, location and depth. J Urol. 2009;182(3):844–53. doi: 10.1016/j.juro.2009.05.035 . 19616235 10.1016/j.juro.2009.05.035

[35] Ficarra V, Novara G, Secco S, Macchi V, Porzionato A, De Caro R, et al Preoperative aspects and dimensions used for an anatomical (PADUA) classification of renal tumours in patients who are candidates for nephron-sparing surgery. Eur Urol. 2009;56(5):786–93. doi: 10.1016/j.eururo.2009.07.040 . 19665284 10.1016/j.eururo.2009.07.040

[36] Owens WD. American Society of Anesthesiologists Physical Status Classification System in not a risk classification system. Anesthesiology. 2001;94(2):378 . 11176115 10.1097/00000542-200102000-00042

