# Supplementary figures and images for: Survival outcomes in patients with large (≥7cm) clear cell renal cell carcinomas treated with nephron-sparing surgery versus radical nephrectomy: Results of a multicenter cohort with long-term follow-up
Source: PLoS One. 2018 May 3;13(5):e0196427. doi: 10.1371/journal.pone.0196427 (PMC5933746; doi:10.1371/journal.pone.0196427)

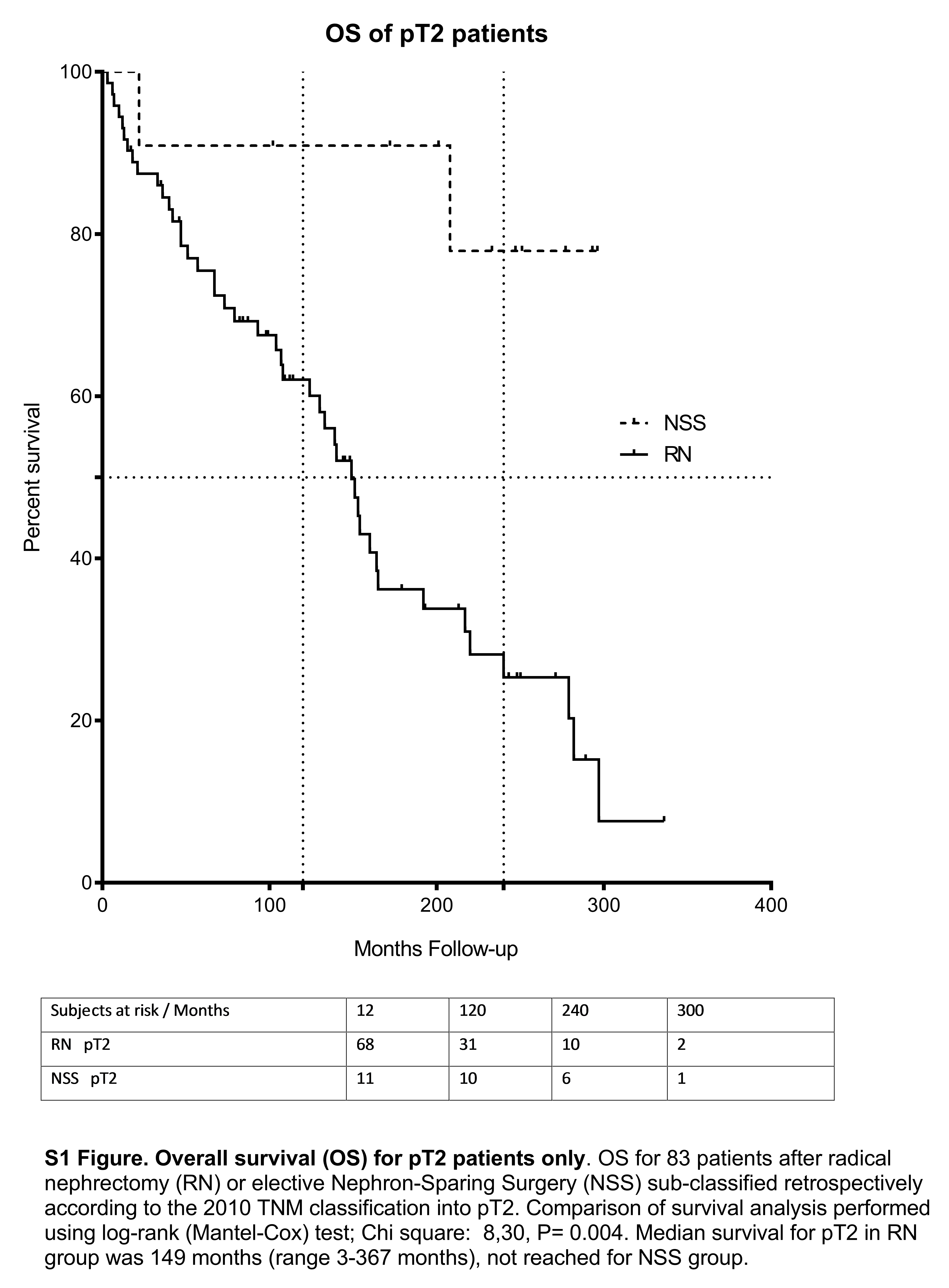

Supplement: S1 Fig — Overall survival (OS) for 83 patients after radical nephrectomy (RN) or elective Nephron-Sparing Surgery (NSS) sub-classified retrospectively according to the 2010 TNM classification into pT2. Comparison of survival analysis performed using log-rank (Mantel-Cox) test; Chi square: 8,30, P = 0.004. Median survival for pT2 in RN group was 149 months (range 3–367 months), not reached for NSS group. (TIF) [file pone.0196427.s001.tif]

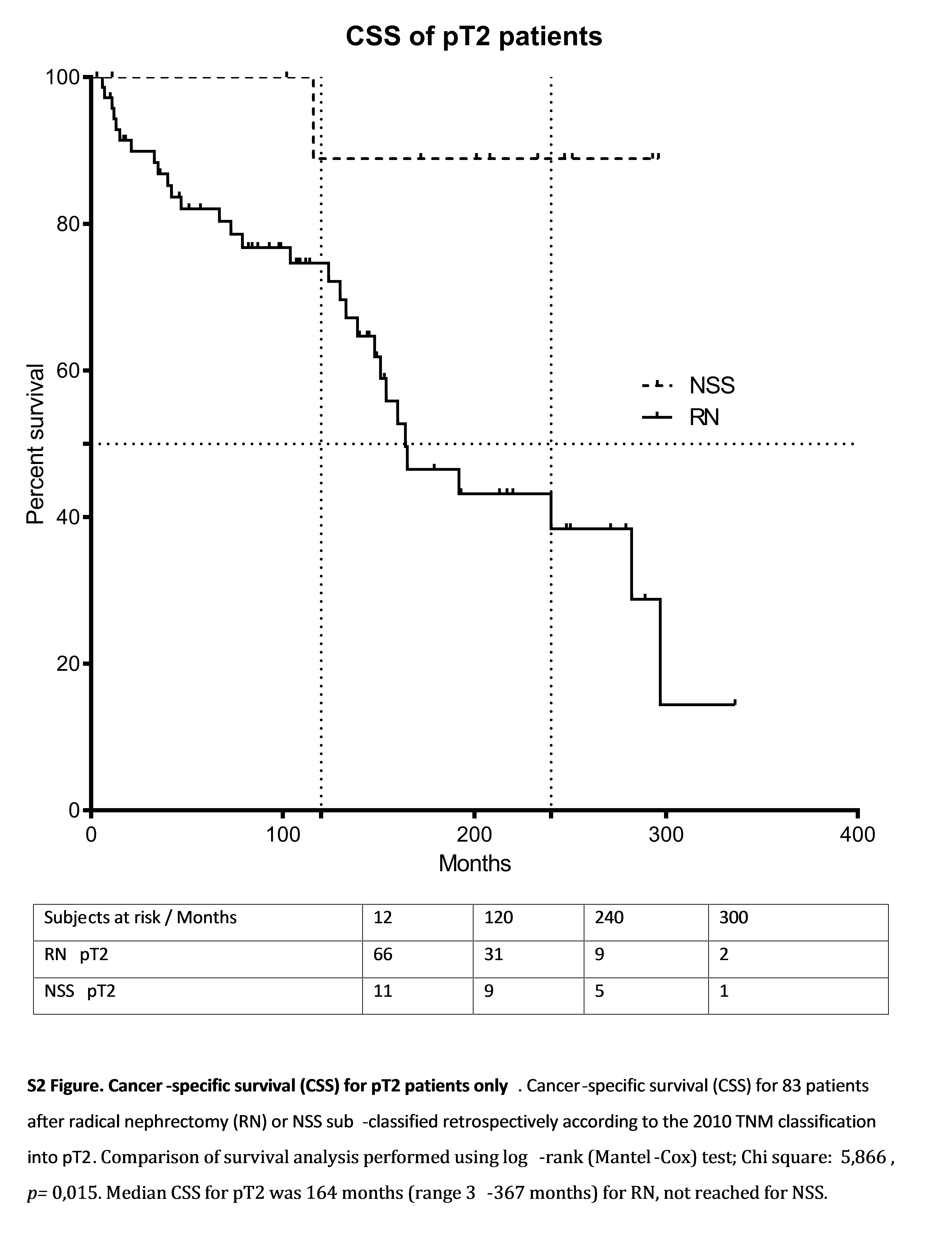

Supplement: S2 Fig — CSS for 83 patients after radical nephrectomy (RN) or NSS sub-classified retrospectively according to the 2010 TNM classification into pT2. Comparison of survival analysis performed using log-rank (Mantel-Cox) test; Chi square: 5,866, p = 0,015. Median CSS for pT2 was 164 months (range 3–367 months) for RN, not reached for NSS. (TIF) [file pone.0196427.s002.tif]

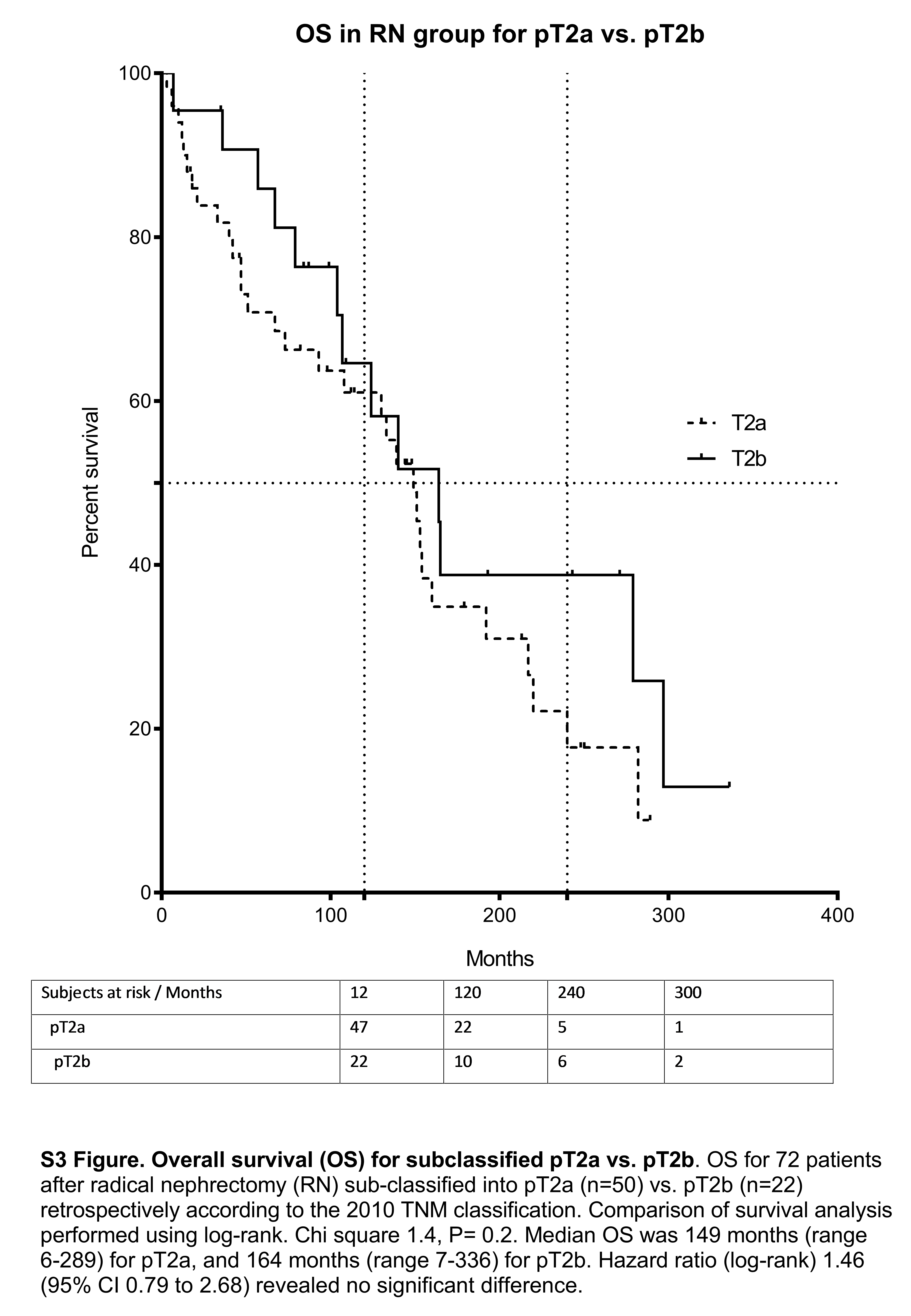

Supplement: S3 Fig — OS for 72 patients after radical nephrectomy (RN) sub-classified into pT2a (n = 50) vs. pT2b (n = 22) retrospectively according to the 2010 TNM classification. Comparison of survival analysis performed using log-rank. Chi square 1.4, P = 0.2. Median OS was 149 months (range 6–289) for pT2a, and 164 months (range 7–336) for pT2b. Hazard ratio (log-rank) 1.46 (95% CI 0.79 to 2.68) revealed no significant difference. (TIF) [file pone.0196427.s003.tif]

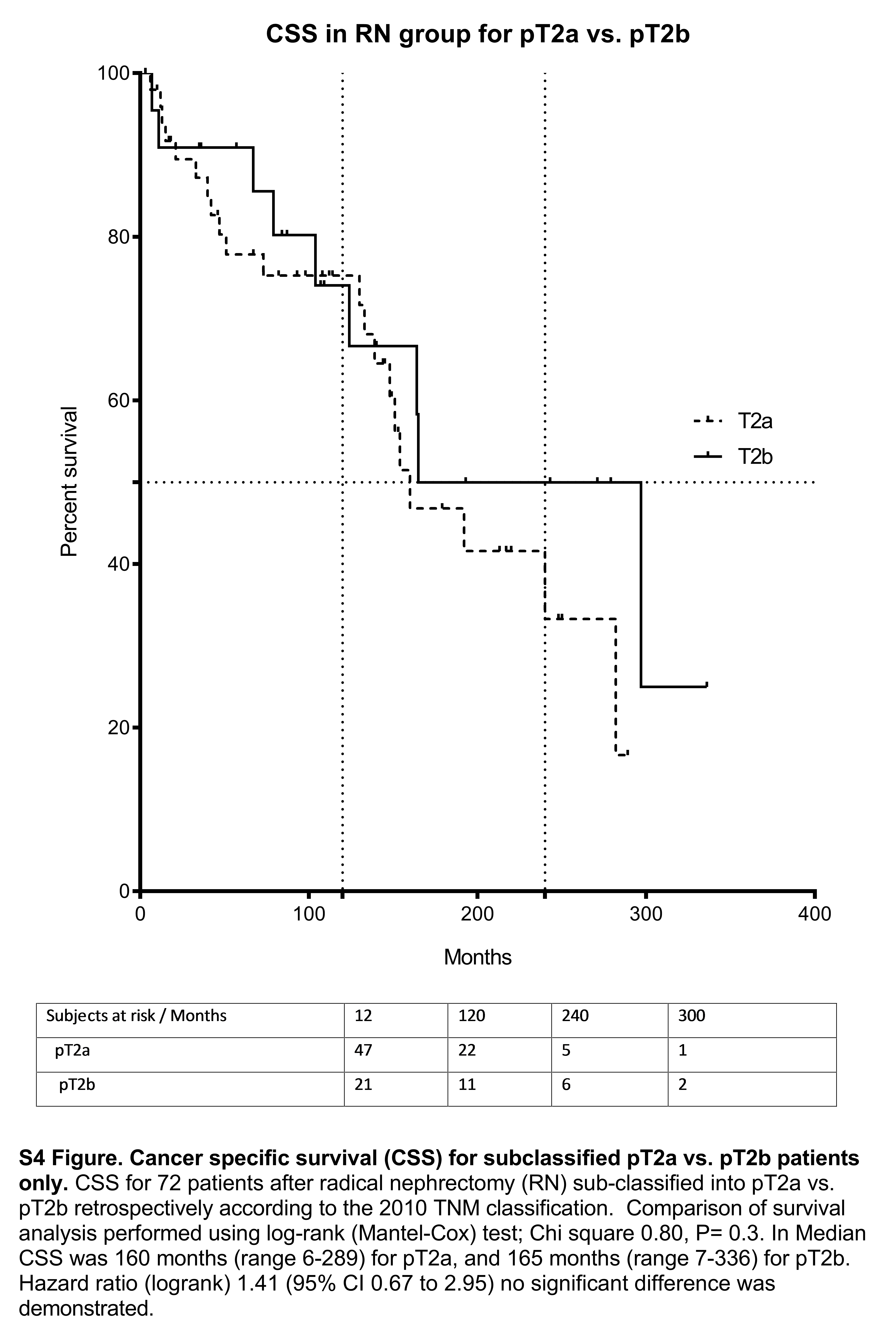

Supplement: S4 Fig — CSS for 72 patients after radical nephrectomy (RN) sub-classified into pT2a vs. pT2b retrospectively according to the 2010 TNM classification. Comparison of survival analysis performed using log-rank (Mantel-Cox) test; Chi square 0.80, P = 0.3. In Median CSS was 160 months (range 6–289) for pT2a, and 165 months (range 7–336) for pT2b. Hazard ratio (logrank) 1.41 (95% CI 0.67 to 2.95) no significant difference was demonstrated. (TIF) [file pone.0196427.s004.tif]

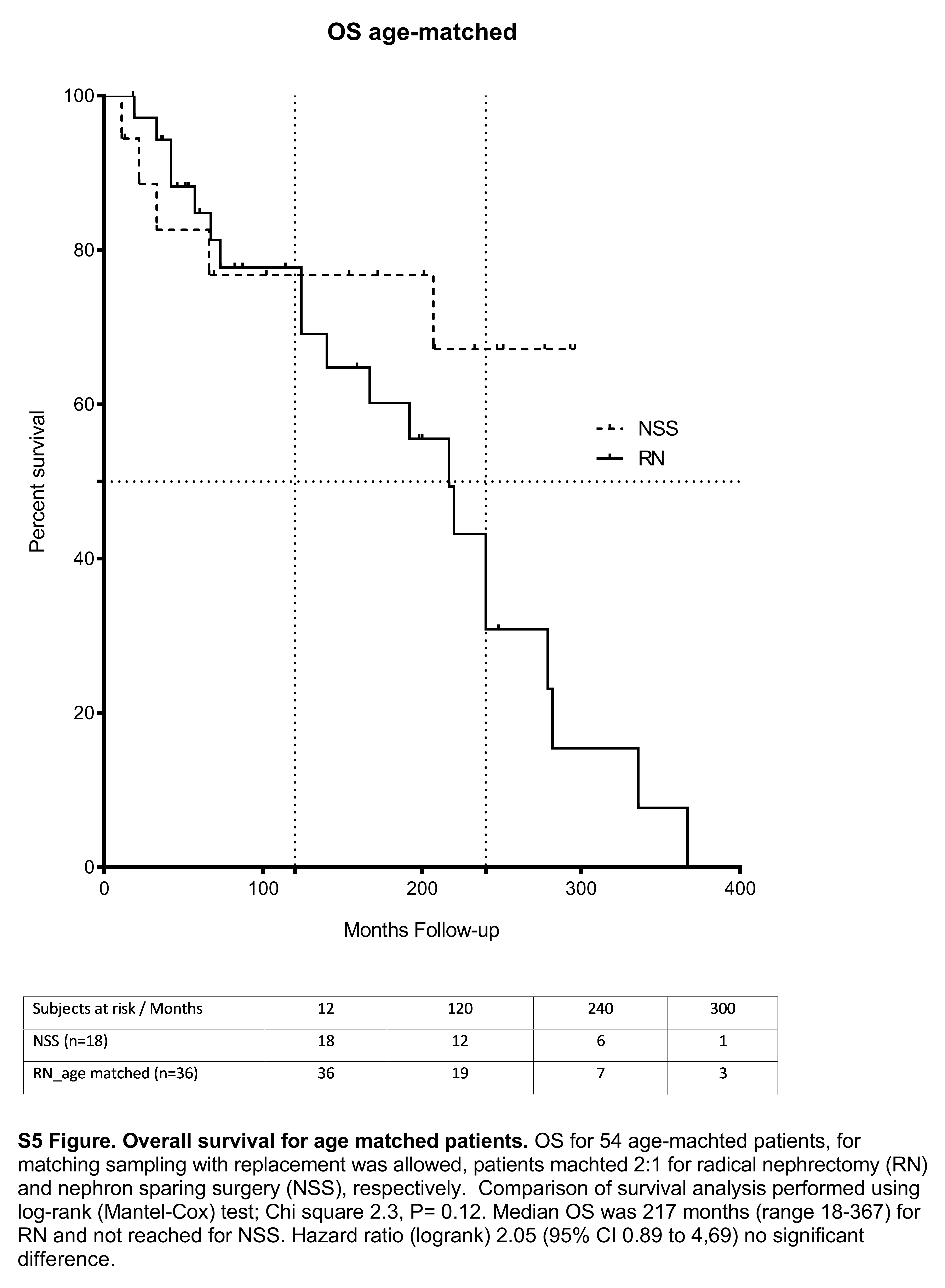

Supplement: S5 Fig — OS for 54 age-machted patients, for matching sampling with replacement was allowed, patients machted 2:1 for radical nephrectomy (RN) and nephron sparing surgery (NSS), respectively. Comparison of survival analysis performed using log-rank (Mantel-Cox) test; Chi square 2.3, P = 0.12. Median OS was 217 months (range 18–367) for RN and not reached for NSS. Hazard ratio (logrank) 2.05 (95% CI 0.89 to 4,69) no significant difference. (TIF) [file pone.0196427.s005.tif]

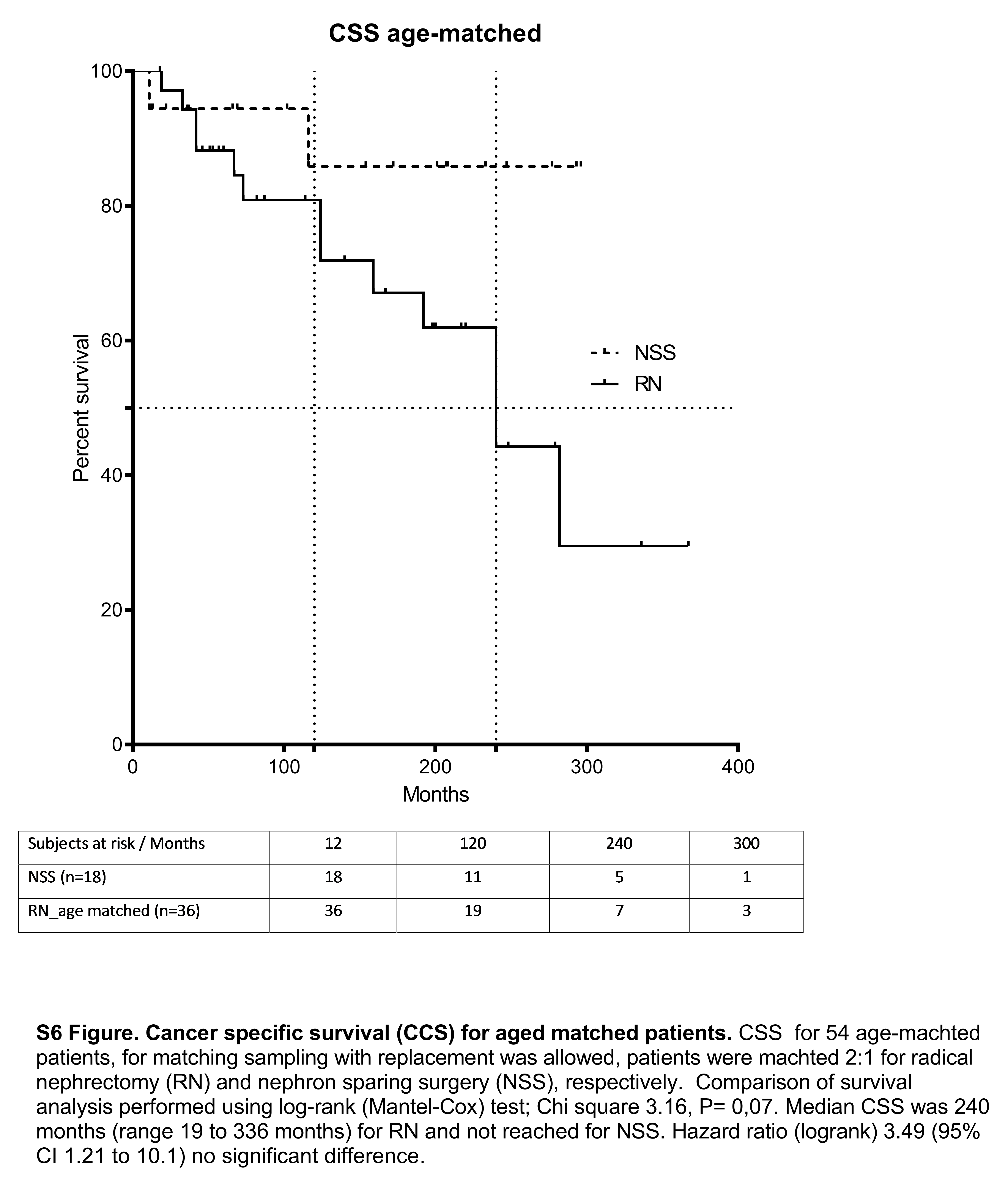

Supplement: S6 Fig — CCS for 54 age-machted patients, for matching sampling with replacement was allowed, patients were machted 2:1 for radical nephrectomy (RN) and nephron sparing surgery (NSS), respectively. Comparison of survival analysis performed using log-rank (Mantel-Cox) test; Chi square 3.16, P = 0,07. Median CSS was 240 months (range 19 to 336 months) for RN and not reached for NSS. Hazard ratio (logrank) 3.49 (95% CI 1.21 to 10.1) no significant difference. (TIF) [file pone.0196427.s006.tif]
